# Cardiovascular morbidities in postoperative colorectal cancer patients

**DOI:** 10.1038/s41598-021-00735-3

**Published:** 2021-11-01

**Authors:** Hyangkyoung Kim, In Ja Park, Youngjin Han, Tae-Won Kwon, Yong-Pil Cho

**Affiliations:** 1 Division of Vascular Surgery, Department of Surgery, University of Ulsan College of Medicine and Asan Medical Center, Seoul, Republic of Korea; 2 Division of Colorectal Surgery, Department of Surgery, Department of Colon and Rectal Surgery, University of Ulsan College of Medicine and Asan Medical Center, 88 Olympic-ro 43-gil, Songpa-gu, Seoul, 05505 Republic of Korea

**Keywords:** Diseases, Oncology

## Abstract

This retrospective observational study investigated the long-term prevalence of new-onset cardiovascular disease (CVD) and the predictive role of atherosclerotic plaque in the aorta and iliac arteries for CVD in postoperative colorectal cancer (CRC) patients who received surgical treatment between 2014 and 2015. CVD included coronary or cerebrovascular diseases which required treatment and new-onset CVD included peri-and postoperatively diagnosed CVDs or aggravated CVDs that required additional treatment during follow-up. Of the 2,875 patients included in this study, the prevalence of CVD was 8.9% (255/2875) and 141 (4.9%) developed new-onset CVD. Maximum arterial stenosis in the aorta or iliac arteries occurred in 40.8 ± 18.6% of patients with new-onset CVD and 11.6 ± 13.8% of patients without new-onset CVD (p < 0.001). The mean new-onset CVD-free survival time in patients with > 30% and < 30% stenoses were 52.5 [95% confidence intervals (CIs) 50.0–54.9] and 66.5 (95% CIs 66.2–66.8) months, respectively (p < 0.001). The area under the receiver operating characteristic curve of the maximal arterial stenosis for new-onset CVD was 0.911. These results suggest that CRC patients are at risk for developing new-onset CVD, which is associated with reduced survival. Atherosclerotic burden in the aorta or both iliac arteries may help predict future CVD events.

## Introduction

Cancer and cardiovascular disease (CVD) are the major causes of mortality and chronic disease burden worldwide^1^. Early diagnosis and advancements in treatment have significantly improved cancer survival rate in recent years^2,3^. Hence, with the increasing age of cancer survivors because of enhanced life expectancy, the overlap between heart disease and cancer is more prominently observed^4^. CVD is emerging as a serious cause of death in many cancer survivors, rivaling cancer recurrence^5^. Although a few conflicting results have been published based on previous studies^6,7^, most cancers are associated with an increased risk of coronary heart disease (CHD), especially during the first 6 months after diagnosis^8^. Emerging evidence have suggested a relationship between CVD and cancer, in addition to simple collusion due to the sharing of common risk factors^9,10^. Chronic inflammation and oxidative stress, known as the underlying pathophysiology for both diseases, are thought to be the major mechanisms linking the two^8^. Other suggested mechanisms are the cardiotoxic effects of systemic therapy, common molecular pathways involving both the diseases, and innate immunity^11–14^.

Colorectal cancer (CRC) is the third most commonly diagnosed cancer globally, accounting for 11% of all cancer diagnoses^15^. In patients with colon cancer, non-cancer mortality is approximately 50% higher than that in the normal population, and CVD is reported in a large proportion of these patients^16^. However, the association between CVD and CRC is not fully characterized, and postoperative surveillance is predominantly focused on cancer recurrence, disconnected from CVD. We aimed to investigate the long-term prevalence of CVD and the predictive role of atherosclerotic plaque in the aorta and iliac arteries for CVD events in postoperative CRC patients.

## Results

### Characteristics of the population

Baseline demographic data of the study population are summarized in Table 1. We excluded 265 of the 3140 patients based on the inclusion criteria. Thus, 2875 patients were included in the study, of which, 1727 (60.1%) were men, and 1148 (39.9%) were women. The median age of the patients was 62 years (IQR 54–70 years). When comparing patients with new-onset CVD and without new-onset CVD, patients with new-onset CVD were significantly older than those without. Higher rate of hypertension, diabetes, kidney disease, and smoker or higher ASA score or ECOG score were observed in the group with new-onset CVD. A total of 170 (5.9%) patients had a history of any type of CVD; 116 (4.0%) patients had a history of coronary artery disease (CAD), and 54 (1.9%) patients had a history of ischemic stroke. Preoperative chemoradiotherapy and postoperative adjuvant chemotherapy were performed in 382 (13.3%) and 1369 (47.6%) patients, respectively (Table 2). Adjuvant radiotherapy was performed in 50 (2.5%) rectal cancer patients. The chemotherapeutic regimens used were as follows: 5-FU/Leucovorin, 148 (5.1%) patients; capecitabine, 288 (10.0%) patients; FOLFOX, 487 (16.9%) patients; XELOX, 192 (6.7%) patients; FOLFIRI, 9 (0.3%) patients; and others, 245 (8.6%) patients. In total, 198 (6.9%) patients died during the follow-up period. The median follow-up duration was 47.7 months (IQR 37.8–55.7 months). The mean survival time was 63.6 months (95% CI 63.0–64.1 months), and the 5-year overall survival rate was 92.2%.

**Table 1:** Baseline clinical characteristics of the patients.

	Study population (N = 2875)	Patients with new-onset CVD (n = 141)	Patients without new-onset CVD (n = 2734)	p
**Age, median (IQR)**	62.0 (IQR, 54.0–70.0)	70.0 (IQR, 61.0–75.0)	61.0 (IQR, 53.0–70.0)	< 0.001
**Age > 65 years, n (%)**	1138 (39.6)	90 (63.8)	1048 (38.3)	< 0.001
**Sex, male (%)**	1727 (60.1)	104 (73.8)	1623 (59.4%)	0.001
**BMI, median (IQR)**	23.5 (IQR, 21.5–25.55)	24.0 (IQR, 21.85–25.8)	23.5 (21.5–25.5)	0.572
**Hypertension, n (%)**	1094 (38.1)	85 (60.3)	1009 (36.9)	< 0.001
**Diabetes mellitus, n (%)**	512 (17.8)	59 (41.8)	453 (16.6)	< 0.001
**Hyperlipidemia, n (%)**	269 (9.4)	11 (7.8)	258 (9.4)	0.656
**Coronary arterial disease, n (%)**	120 (4.2)	49 (34.8)	71 (2.6)	< 0.001
**Pulmonary disease, n (%)**	141 (4.9)	8 (5.7)	133 (4.9)	0.687
**Ischemic stroke, n (%)**	61 (2.1)	11 (7.8)	50 (1.8)	< 0.001
**Valvular heart disease, n (%)**	701 (24.4)	39 (27.6)	662 (24.2)	0.366
**Atrial fibrillation, n (%)**	31 (1.1)	5 (3.5)	26 (1.0)	0.016
**Chronic kidney disease, n (%)**	59 (2.1)	12 (8.5)	47 (1.7)	< 0.001
**ASA, n (%)**				
1	934 (32.5)	10 (7.1)	924 (33.8)	< 0.001
2	1794 (62.4)	76 (53.9)	1718 (62.8)	
3	126 (4.4)	46 (32.6)	80 (2.9)	
4	21 (0.7)	9 (6.4)	12 (0.4)	
**ECOG, n (%)**				
1	2659 (92.5)	102 (72.3)	2557 (93.5)	< 0.001
2	206 (7.2)	34 (24.1)	172 (6.3)	
3	8 (0.3)	3 (2.1)	5 (0.2)	
4	2 (0.1)	2 (14)	0	
**Smoking, n (%)**				
Current smoker	247 (8.6)	19 (13.5)	228 (8.3)	0.003
Ex-smoker	1006 (35.0)	56 (39.7)	950 (34.7)	
**Alcohol, n (%)**				
Current drinker	515 (17.9)	33 (23.6)	482 (17.6)	0.247
Ex-drinker	936 (32.6)	40 (28.6)	896 (32.8)	
**Total cholesterol, mean (SD)**	153.09 (35.0)	141.3 (37.7)	153.7 (34.8)	< 0.001
**CRP, mean (SD)**	0.48 (1.64)	0.68 (1.79)	0.46 (1.60)	0.190

*CVD* cardiovascular disease, *IQR* interquartile range, *BMI* body mass index, *ASA* American Society of Anesthesiologists Physical Status Classification System, *ECOG* Eastern Cooperative Oncology Group performance status, *SD* standard deviation, *CRP* c-reactive protein.

**Table 2:** Clinical characteristics of the patients.

	Study population (N = 2875)	Patients with new-onset CVD (n = 141)	Patients without new-onset CVD (n = 2734)	p
**Tumor location, n (%)**				
Colon	892 (31.0)	42 (29.8)	850 (31.1)	0.780
Rectum	1983 (69.0)	99 (70.2)	1884 (68.9)	
**Stage, n (%)**				
0	136 (4.7)	6 (4.3)	130 (4.8)	0.874
I	760 (26.4)	33 (23.4)	727 (26.6)	
II	945 (32.9)	54 (38.3)	891(32.6)	
III	1030 (35.8)	48 (34.0)	982 (35.9)	
Unknown	4 (0.1)	0 (0)	4 (0.1)	
**Adjuvant chemotherapy, n (%)**	1369 (47.6)	51 (36.2)	1318 (48.2)	0.006
**Adjuvant radiotherapy, n (%)**^**a**^	76 (3.8)	7 (57.1)	69 (3.7)	0.104

^a^Frequency was calculated in patients with rectal cancer.

### Prevalence of CVDs and the risk factors

The prevalence of CVD in the study cohort was 8.9% (255/2875). Of the 170 patients with a history of any type of preoperative CVD, 56 (32.9%) experienced worsening of CVDs, whereas 114 (67.1%) did not exhibit any symptom or sign. A total of 141 (4.9%) patients developed new-onset CVDs, of which, 56 (39.7%) required treatment due to aggravation of preoperative CVD, and 85 were newly diagnosed with CVD. The most common type of CVD was CAD (n = 91, 64.5%), followed by ischemic stroke (n = 50, 35.5%). Two (1.4%) patients experienced a CVD event within 3 days prior to the operation. Fifteen (10.6%) patients developed new-onset CVD within 1 month post operation. Within 2 years, 79 (56.0%) patients developed new-onset CVD. Among the patients with new-onset CVD, the rate of new-onset CVD was higher among those aged > 65 years than those aged < 65 years (7.9% [90/1138] vs. 2.9% [51/1737], p < 0.001). Among the 2,677 survivors, the prevalence of new-onset CVD was 7.4% in those aged > 65 years (75/1010) and 2.8% in those aged < 65 years (47/1667).

Out of the 141 patients with new-onset CVD, 19 (13.5%) died during the follow-up period. The mean new-onset CVD-free survival time was 64.7 (64.3–65.2) months, and the 5-year new-onset CVD-free survival rate was 94.5%. The risk factors associated with new-onset CVD were analyzed (Table 3). General atherosclerotic risk factors and factors that affect CVD including age, sex, hypertension, diabetes, previous history of coronary arterial disease or ischemic stroke, smoking, ASA score, chemotherapy, and hypercholesterolemia were included in the analysis for adjustment. After adjusting for these potential confounders, old age (HR 1.044 [95% CI 1.024–1.063], p < 0.001), diabetes mellitus (HR 1.712 [95% CI 1.197–2.449], p = 0.003), current smoker (HR 2.109 [95% CI 1.234–3.606], p = 0.006), ASA 2 (HR 2.544 [95% CI 1.231–5.255], p = 0.012), ASA 3 (HR 10.026 [95% CI 4.084–24.615], p < 0.001), and ASA 4 (HR 16.598[95% CI 6.055–45.501], p < 0.001) were found to be related to the increased risk of new-onset CVD. A subgroup analysis according to whether the tumor was in the colon or rectum is summarized in the Table 4.

**Table 3:** Independent predictors of new-onset cardiovascular diseases in the Cox regression model.

	Unadjusted HR			Adjusted HR		
	HR	95% CI	p	HR	95% CI	p
Age	1.073	1.056–1.091	< 0.001	1.044	1.024–1.063	< 0.001
Female sex	0.524	0.360–0.763	0.001			
Hypertension	2.736	1.952–3.836	< 0.001			
DM	3.547	2.538–4.958	< 0.001	1.712	1.197–2.449	0.003
CKD	4.848	2.683–8.762	< 0.001			
Previous CAD	14.669	10.356–20.776	< 0.001	3.055	1.789–5.215	< 0.001
Previous ischemic stroke	5.316	2.870–9.845	< 0.001			
Non-smoker			0.023			0.024
Current smoker	1.952	1.171–3.255	0.010	2.109	1.234–3.606	0.006
Ex-smoker	1.381	0.966–1.975	0.077	1.188	0.824–1.714	0.356
ASA			< 0.001			< 0.001
ASA (2)	4.271	2.209–8.259	< 0.001	2.544	1.231–5.255	0.012
ASA (3)	51.339	25.850–101.958	< 0.001	10.026	4.084–24.615	< 0.001
ASA (4)	55.002	22.329–135.483	< 0.001	16.598	6.055–45.501	< 0.001
Chemotherapy	0.551	0.391–0.777	0.001			
Hypercholesterolemia	0.808	0.436–1.494	0.496			

*ASA* American Society of Anesthesiologists Physical Status Classification System, *BMI* body mass index, *CAD* coronary arterial disease, *CI* confidence interval, *CKD* chronic kidney disease, *DM* diabetes mellitus, *HR* hazard ratio.

**Table 4:** Subgroup analysis of the independent predictors of new-onset cardiovascular diseases in patients with colon and rectal cancer.

	Unadjusted HR			Adjusted HR		
	HR	95% CI	p	HR	95% CI	p
**Colon cancer**						
Age	1.082	1.050–1.115	< 0.001	1.050	1.016–1.086	0.004
Female sex	1.004	0.547–1.843	0.990			
Hypertension	4.473	2.288–8.744	< 0.001			
DM	3.485	1.890–6.427	< 0.001			
CKD	0.986	0.136–7.177	0.989			
Previous CAD	12.191	6.446–23.055	< 0.001	3.986	1.554–10.229	0.004
Previous ischemic stroke	2.597	0.627–10.754	0.188			
Non-smoker			0.808			
Current smoker	1.417	0.493–4.072	0.518			
Ex-smoker	1.016	0.510–2.022	0.965			
ASA			< 0.001			0.004
ASA (2)	3.149	1.085–9.139	0.035	1.938	0.641–5.859	0.241
ASA (3)	27.217	8.750–84.654	< 0.001	4.527	1.028–19.925	0.046
ASA (4)	61.656	15.224–249.705	< 0.001	14.843	3.087–71.382	0.001
Chemotherapy	0.369	0.176–0.772	0.008			
Hypercholesterolemia	0.705	0.170–2.919	0.630			
**Rectal cancer**						
Age	1.070	1.049–1.091	< 0.001	1.042	1.018–1.066	< 0.001
Female sex	0.358	0.215–0.596	< 0.001			
Hypertension	2.255	1.516–3.353	< 0.001			
DM	3.543	2.375–5.286	< 0.001	1.840	1.201–2.821	0.005
CKD	7.415	3.961–13.882	< 0.001			
Previous CAD	15.903	10.493–24.101	< 0.001	2.833	1.505–8.332	0.001
Previous ischemic stroke	6.960	3.503–13.830	< 0.001			
Non-smoker			0.015			0.016
Current smoker	2.229	1.231–4.035	0.008	2.515	1.342–4.715	0.004
Ex-smoker	1.563	1.019–2.400	0.041	1.298	0.838–2.011	0.243
ASA			< 0.001			< 0.001
ASA (2)	4.997	2.149–11.616	< 0.001	2.602	1.073–6.310	0.034
ASA (3)	68.671	28.766–163.931	< 0.001	13.591	4.725–39.091	< 0.001
ASA (4)	49.604	15.115–162.787	< 0.001	15.520	4.303–55.984	< 0.001
Chemotherapy	0.621	0.417–0.927	0.020			
Adjuvant radiotherapy	1.768	0.649–4.1845	0.265			
Hypercholesterolemia	0.835	0.421–1.656	0.605			

*ASA* American Society of Anesthesiologists Physical Status Classification System, *BMI* body mass index, *CAD* coronary arterial disease, *CI* confidence interval, *CKD* chronic kidney disease, *DM* diabetes mellitus, *HR* hazard ratio.

Anastomotic leakage developed in 15 patients (37.5%). Anastomotic leakage was found to be not associated with the new-onset CVD (p = 0.334). Inferior mesenteric artery (IMA) occlusion did not occur in any of the patients with anastomotic leakage.

### Relationship between degree of arterial stenosis and CVD

The degree of stenosis in the aorta and bilateral iliac arteries are summarized in Table 5. The degree of arterial stenosis was significantly higher in patients with new-onset CVD than in those without CVD (aorta: 30.4% ± 9.5% vs. 8.2% ± 11.0%; RCIA: 28.5% ± 9.1% vs. 6.5% ± 11.7%; LCIA: 26.2% ± 20.3% vs. 5.7% ± 11.7%; and SMA: 12.1% ± 21.3% vs. 0.7% ± 4.6%; all p < 0.001). The maximal degree of stenosis anywhere in the aorta or iliac arteries was 40.8% ± 18.6% in patients with new-onset CVD and 11.6% ± 13.8% in patients without CVD (p < 0.001). The IMA occlusion rate was significantly higher in patients with new-onset CVD than in those without CVD (37 [26.2%] vs. 53 [1.9%]; p < 0.001). Moreover, the proportion of patients with > 30% stenosis was significantly higher in those with new-onset CVD than in those without CVD (98 [69.5%] vs. 282 [10.3%]; p < 0.001). The maximal degree of arterial stenosis was significantly higher in patients aged > 65 years than in those aged < 65 years (20.7% ± 15.9% vs 8.4% ± 13.4%, p < 0.001). Only 1 (0.1%) of the 1,393 patients without atherosclerotic plaque anywhere in the aorta and both iliac arteries developed new-onset CVD.

**Table 5:** Degree of arterial stenosis.

	New-onset CVD (n = 162)	No CVD (n = 2713)	p
Aorta, %	30.4 ± 9.5	8.2 ± 11.0	< 0.001
RCIA, %	28.5 ± 9.1	6.5 ± 11.7	< 0.001
LCIA, %	26.2 ± 20.3	5.7 ± 11.7	< 0.001
SMA, %	12.1 ± 21.3	0.7 ± 4.6	0.001
Any location, maximal stenosis, %	40.8 ± 18.6	11.6 ± 13.8	< 0.001
IMA occlusion	37 (26.2%)	53 (1.9%)	< 0.001
Aorta, > 50% stenosis	7 (5.0%)	2 (0.1%)	< 0.001
Iliac artery, > 50% stenosis	25 (17.7%)	28 (1.0%)	< 0.001
Any location, > 20% stenosis	130 (92.2%)	829 (30.3%)	< 0.001
Any location, > 30% stenosis	98 (69.5%)	282 (10.3%)	< 0.001
Any location, > 50% stenosis	32 (22.7%)	31 (1.1%)	< 0.001

*CVD* cardiovascular disease, *IMA* inferior mesenteric artery, *LCIA* left common iliac artery, *RCIA* right common iliac artery, *SMA* superior mesenteric artery.

**Figure 1:**
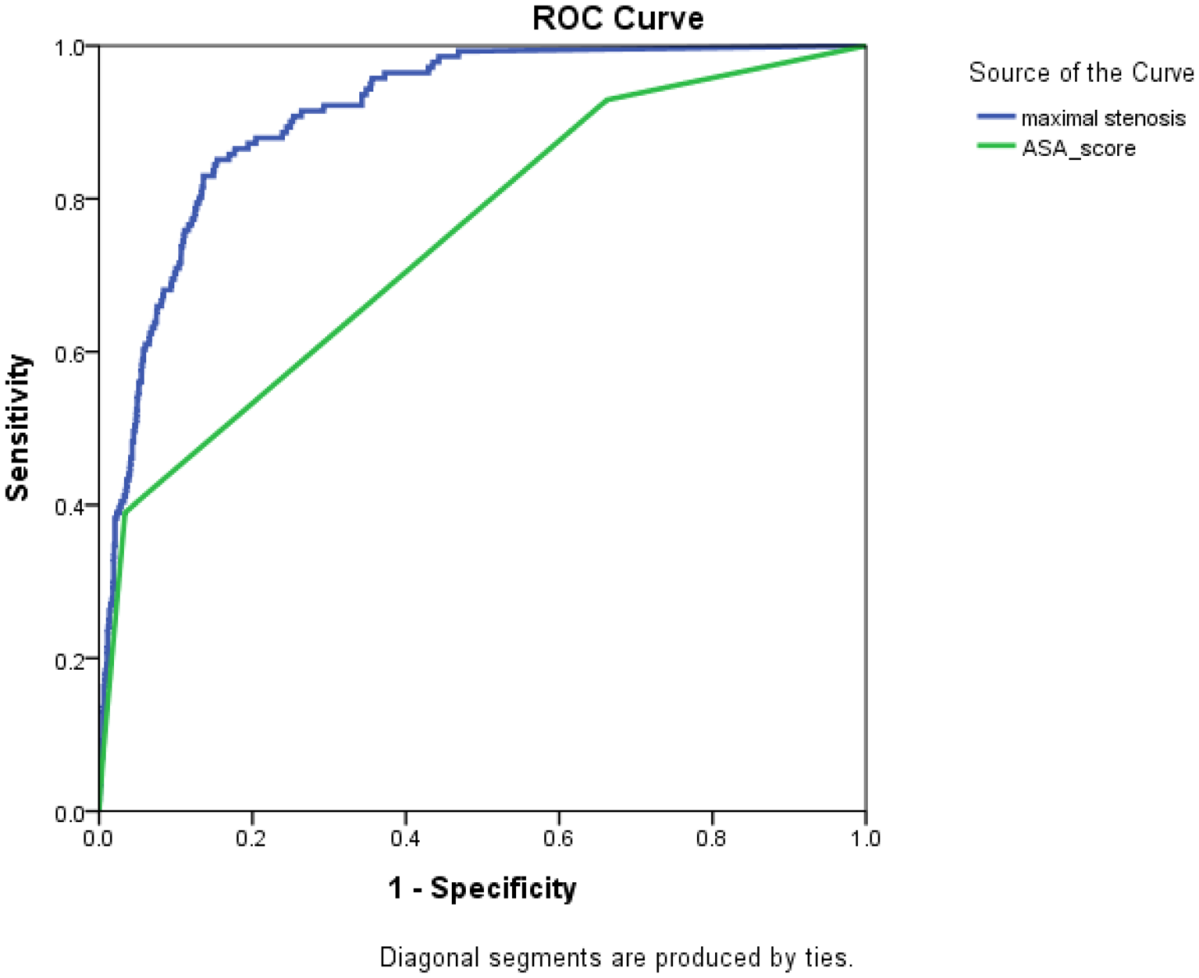
Receiver operating characteristic curve of the arterial stenosis degree for new-onset cardiovascular disease. The area under the curve of the ASA score is 0.747 and 0.911 for the maximal arterial stenosis. *ASA* American Society of Anesthesiologists Physical Status Classification System.

**Figure 2:**
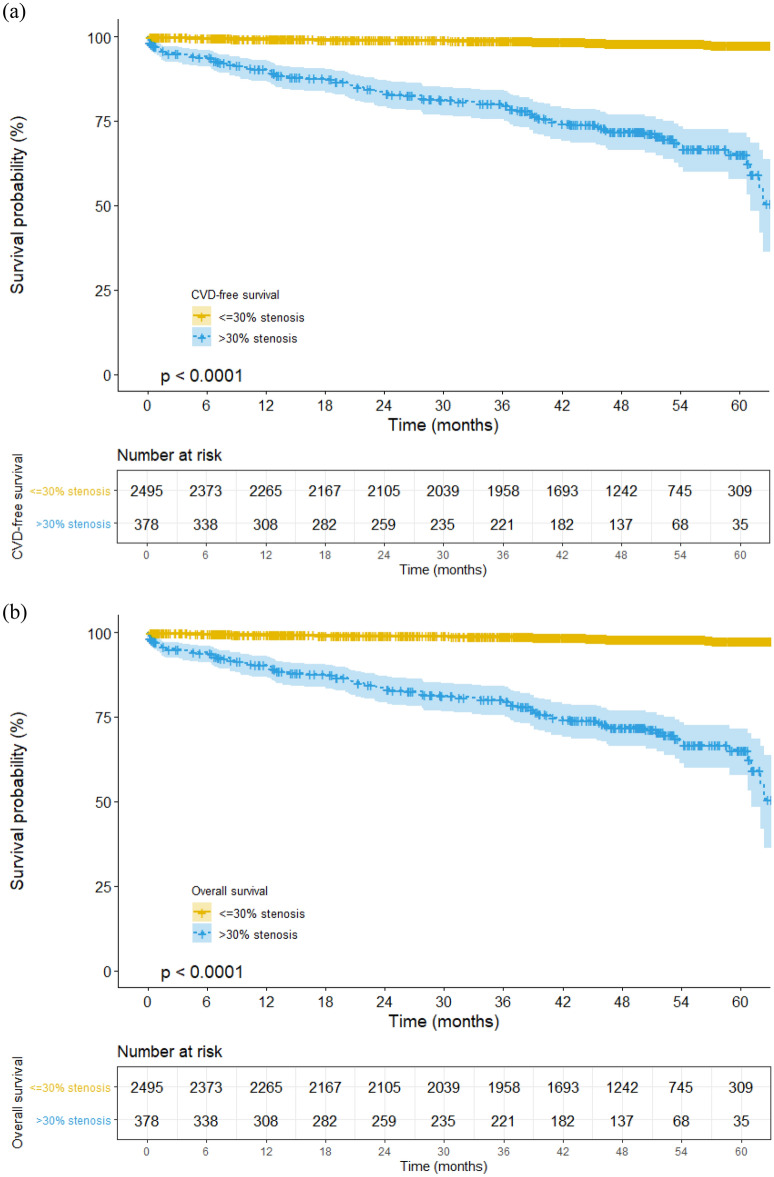
Cumulative survival rate/time. (**a**) New-onset cardiovascular disease-free survival rate and (**b**) overall survival rate.

The ROC curve of the degree of arterial stenosis for new-onset CVD is depicted in Fig. 1. The area under the curve (AUC) of maximal arterial stenosis was 0.911 and 0.747 for the ASA score. CVD-free and overall survival time were compared in those with > 30% and < 30% stenosis (Fig. 2). The mean CVD-free survival time was 52.5 (95% CI 50.0–54.9) months in patients with > 30% stenosis and 66.5 (95% CI 66.2–66.8) months in those with < 30% stenosis (p < 0.001, Fig. 2a). The mean overall survival time was 59.7 (95% CI 57.8–61.6) months in patients with > 30% stenosis and 64.1 (95% CI 63.6–64.6) months in those with < 30% stenosis (p < 0.001, Fig. 2b). The predictability of > 30% arterial stenosis for new-onset CVD were as follows: sensitivity, 69.5% (98/141); specificity, 89.7% (2452/2734); accuracy, 88.7% ([98 + 2452]/2875); positive predictive value, 25.8% (98/380); and negative predictive value, 98.3% (2452/2495). The predictability of > 20% arterial stenosis for new-onset CVD were as follows: sensitivity, 92.2% (130/141); specificity, 69.7% (1905/2734); accuracy, 70.8% ([130 + 1905]/2875); positive predictive value, 13.6% (130/959); and negative predictive value, 99.4% (1905/1916). Patients with > 30% stenosis had 19.8 times higher risk of new-onset CVD than those with < 30% stenosis (95% CI 13.6–29.0, p < 0.001). The maximal degree of arterial stenosis was significantly associated with the development of new-onset CVD (coefficient 1.099 [95% CI 1.085–1.113]; p < 0.001). In addition, IMA occlusion was significantly associated with new-onset CVD (coefficient 17.997 [11.323–28.603]; p < 0.001).

## Discussion

Among the existing cancer-related comorbidities, cardiovascular risks have a profound effect on the health of cancer patients. The bidirectional link between cancer and CVD has been proposed in both experimental and clinical studies. A study using a mouse model for ischemic cardiomyopathy suggested a causal link between heart failure and colon cancer, with increased susceptibility to intestinal tumor^17^. In a large clinical study, older adults with stage I to III CRC were found to have a substantial risk of new-onset CVD^18^. Therefore, a proactive approach toward early detection and proper risk factor management is required.

Atherosclerosis is a systemic disease and affects numerous vascular beds. The prevalence of CHD in patients with PAOD ranges from 46 to 71%^19,20^. In addition, PAOD is a marker of systemic atherosclerosis, and it increases the risk for future atherosclerotic cardiovascular events^21^. Patients with PAOD may have more extensive atherosclerosis than those with CHD or cerebrovascular disease. Moreover, the occurrence of atherosclerosis in multiple vascular beds such as the leg, heart, and brain is associated with a higher risk of future CVD events^22,23^. In this regard, we evaluated whether PAOD in the abdominopelvic vasculatures has any clinical significance as a surrogate marker for CVD. In the present study, the risk of occurrence of CVD events increased linearly with the degree of arterial stenosis. The survival rate in patients with ≥ 30% stenosis was worse than in those without stenosis.

In this study, the prevalence of new-onset CVD was 4.9%. The prevalence of CVD in survivors aged > 65 years was 7.9%, while 2.9% of patients aged < 65 years developed new-onset CVD. The prevalence of new-onset CVD in survivors aged > 65 years was significantly lower in this study than in a previous report comprising a 10-year cumulative incidence^23^. This discrepancy might be due to the differences in CVD definition and target population, as well as follow-up duration. We did not consider chemotherapy-induced congestive heart failure and valvular heart disease as CVDs because their mechanism is disparate. We also included deceased patients in the analysis because it was not possible to acquire information regarding the cause of death of all patients, some of which died from CVD.

Previous literature suggested a linkage between CVD and cancer, in that both have shared risk factors or common biological mechanisms^18,24–26^. CVD is also expected to affect long-term survival in cancer patients^27,28^. Unsurprisingly, our study showed that patients with new-onset CVDs exhibited worse baseline cardiovascular risk factors (Tables 1 and 3). In the adjusted model, increased age, DM, previous CAD, current smoking, and high ASA score were independent predictors for new-onset CVD. Therefore, it seems useful to evaluate the baseline CVD risk profile in CRC patients. Interestingly, only 32.9% of patients with preoperative CVD had a new CVD event. In this context, the AUC of the ASA score based on prior history was found to be low, implying that it may be inappropriate to limit the screening of patients based only on their history of CVD. However, because performing additional examinations in all patients is inefficient, the importance of specifying patients at risk of CVD is not controversial. In general, several options have been proposed for the early diagnosis and treatment of CVD. One of the most widely used tools is the Framingham risk score, which is calculated based on the patient’s age, gender, blood pressure, and social status^29^. In addition, the ankle-brachial pressure index (ABI), coronary artery calcium scoring, and carotid intima-media thickness (IMT) are used for the early diagnosis of CVD^30–32^. These tools, however, have their corresponding disadvantages and limitations. Coronary calcium scoring has an added risk of radiation exposure, and ABI and carotid IMT require additional testing. The Framingham risk score is relatively simple and useful in predicting future cardiovascular events, but it cannot predict any CVD other than CAD and may under- or overestimate the risk in people whose race are different from majority of the US population^33^. Considering these constraints, this study was designed to suggest an efficient screening method for CVD risk by analyzing the prevalence and risk factors of patients experiencing new-onset CVD events during CRC management and follow-up. In CRC patients with normal kidney function, surveillance of cancer recurrence using enhanced CT is widely performed. If the CVD risk can be assessed with CT, no additional examinations would be required. Hence, we evaluated the utility of detecting the presence of atherosclerotic burden on CT, which is included in the routine surveillance protocol, as a screening tool for CVD. Three cut-off values (i.e., 20%, 30% and 50%) were used to identify the maximal degree of stenosis as a screening test. The cut-off value of 30% was used because the mean value of the degree of maximal stenosis in the aorta and iliac arteries was about 30%. A value of 20% stenosis was used for better sensitivity, while 50% stenosis was used to represent hemodynamic significance. In the present study, the atherosclerotic plaque in the aortoiliac artery exhibited the potential to predict CVD risk in CRC patients. The AUC of the maximal degree of arterial stenosis was 0.911, which was excellent. The sensitivity of 20% stenosis was 92.2%, which showed the possibility of using it as a screening test. On the contrary, 30% stenosis had high specificity but low sensitivity, thus, and it should be combined with other methods that can be easily used clinically. Therefore, it is helpful to predict future new-onset CVD events by checking the atherosclerotic burden in the aorto-iliac arteries on follow-up CT scan in CRC patients, however, the decision on which cut-off value to use might depend on whether the CVD risk assessment has been made in advance.

In this study, we propose the atherosclerotic plaque observed on preoperative CT scan as a surrogate marker for future cardiovascular risk because we thought that it would be more convenient for CVD risk assessment; this approach is more intuitive than traditional CVD risk prediction based on the presence of chronic diseases such as hypertension and DM, and the ASA score. However, we believe that the need to emphasize the importance of CVD risk management is greater than the need to develop an effective method for CVD risk assessment. CVD may occur during long-term follow-up of cancer patients, which may affect the survival^34^.

Another interesting observation (although without statistical significance) was that no patient with anastomotic leakage had IMA occlusion. In other words, contrary to the conventional belief that IMA may be related to anastomotic leakage, leakage occurred only in patients with open IMA. In the lower extremity arteries, the clinical manifestations of acute occlusion are often less dramatic in patients with underlying atherosclerosis than in patients with normal vessels because of already developed collateral circulation. Similarly, it can be postulated that the already occluded IMA does not significantly affect the surgical outcome in the IMA territory.

Our study has important clinical implications as its results showed the prevalence of new-onset CVD in overall CRC patients during the postoperative follow-up period. Another important strength of our study is the relatively large sample size with long-term follow-up, which provided reliable evidence regarding the prevalence of CVD in CRC patients. In addition, this study clearly demonstrated that arterial stenosis is a powerful predictor of new-onset CVD in patients who underwent CRC surgery. Hence, conducting atherosclerotic disease screening for individuals with the highest risk of ischemic disease is desirable and cost-effective. These findings may lead to clinical trials focusing on patients who will most likely benefit from appropriate screening protocols.

This study certainly has some limitations as this is a single-center observational study. Furthermore, since we only used hospital records in the analysis, the lack of information on the cause of death, especially those outside the hospital, may also be an important limitation.

## Conclusions

CRC patients are at risk of developing new-onset CVD, which is associated with reduced survival. It underscores the importance of noncancer-related mortality and CVD surveillance in the management of CRC patients. Atherosclerotic burden in the aorta or both iliac arteries can be considered as one of the predictive tools for future CVD events.

## Methods

A single-center observational study was conducted on patients with CRC who underwent surgery at the Asan Medical Center between January 2014 and December 2015. Patients with primary CRC who underwent curative resection were included in the study. Patients with concurrent distant metastasis at diagnosis, those who did not undergo resection, and those with concurrent or prior malignancies within 5 years of the diagnosis of rectal cancer were excluded. The clinical and imaging data of the included patients were retrospectively reviewed. This study was approved by the Asan medical center Institutional Review Board (No. 2020-0679). The need for informed consent was waived by the Asan medical center Institutional Review Board because of the retrospective nature of this study and lack of information on the participants’ identification. This study complies with the Declaration of Helsinki.

### Study outcomes and definitions

The objective of this study was to evaluate the prevalence of CVDs and new-onset CVDs in CRC patients. The secondary aim was to evaluate whether the detection of aorto-iliac atherosclerosis on preoperative computed tomography (CT) images was associated with CVD occurrence during follow-up. CVD included coronary or cerebrovascular diseases which required treatment and new-onset CVD included peri- or postoperatively diagnosed CVDs or aggravated CVDs that required additional treatment during follow-up.

**Figure 3:**
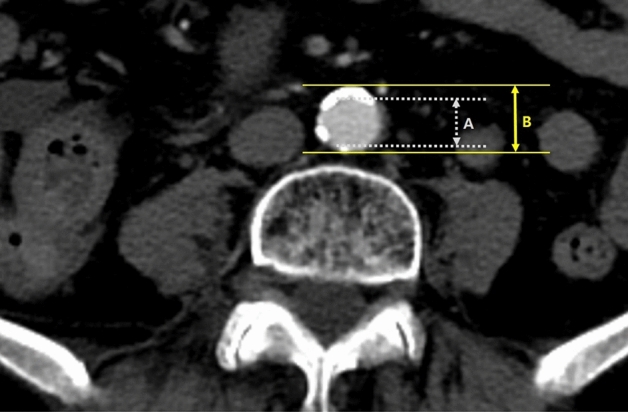
Measurement of the degree of stenosis. The degree of stenosis was calculated using the formula, (B − A)/B × 100.

The prevalence of CVD and new-onset CVD and its risk factors, such as age, sex, hypertension, diabetes, history of CVD, the American Society of Anesthesiologists Physical Status Classification System (ASA) score, chemotherapy, and radiotherapy were evaluated. To investigate whether the detection of the arterial stenosis has a predictive role for these CVDs, we evaluated the images of preoperative enhanced abdominopelvic computed tomography (CT) performed within 1 week before surgery. CT data (slice thickness: 5 mm) of all patients were available. The degree of maximal arterial stenosis was measured at the most severely narrowed segment of the abdominal aorta, superior mesenteric artery (SMA), right common iliac artery (RCIA), and left common iliac artery (LCIA) on CT images. When the plaque was eccentric, maximal arterial stenosis was measured in the narrowest section. The diameter reduction was calculated by dividing the plaque thickness by the arterial diameter (Fig. 3). Maximal stenosis was defined as the highest degree of stenosis anywhere in the aorta or iliac arteries. We also investigated whether anastomotic leakage was related to inferior mesenteric artery (IMA) occlusion.

### Statistical analysis

Descriptive statistics were used to determine the baseline characteristics of the study population and the prevalence of CVDs or postoperative complications. Categorical variables were presented as frequencies or percentages. Continuous variables were presented as mean (standard deviation) or median (interquartile ranges [IQRs]) after performing the normality test (Kolmogorov–Smirnov test). To analyze the associations between the risk factors and new-onset CVD events, variables related to the general CVD risks were fitted in a Cox proportional hazard regression model to determine the unadjusted hazard ratio (HR) and corresponding 95% confidence intervals (CIs). Variables with a p value < 0.1 were selected with backward elimination method to determine the adjusted HR. The association between the degree of arterial stenosis and new-onset CVD was analyzed by logistic regression analysis presented as odds ratios (ORs) and 95% CI. Receiver operating characteristic (ROC) analysis was performed to evaluate the predictability of the degree of stenosis. A p value of < 0.05 was considered statistically significant. All statistical analyses were 2-sided and were performed using SPSS version 21.0 (IBM Corp., Armonk, NY, USA).

## References

[1] Wang H (2016). Global, regional, and national life expectancy, all-cause mortality, and cause-specific mortality for 249 causes of death, 1980–2015: A systematic analysis for the Global Burden of Disease Study 2015. Lancet.

[2] Siegel RL, Miller KD, Jemal A (2020). Cancer statistics, 2020. CA Cancer J. Clin..

[3] Hashim D (2016). The global decrease in cancer mortality: Trends and disparities. Ann. Oncol..

[4] Sturgeon KM (2019). A population-based study of cardiovascular disease mortality risk in US cancer patients. Eur. Heart J..

[5] Henson KE (2016). Cardiac mortality among 200 000 five-year survivors of cancer diagnosed at 15 to 39 years of age: The teenage and young adult cancer survivor study. Circulation.

[6] Shin DW (2020). Risk of ischemic heart disease and stroke in prostate cancer survivors: A Nationwide Study in South Korea. Sci. Rep..

[7] Boekel NB (2016). Cardiovascular disease risk in a large, population-based cohort of breast cancer survivors. Int. J. Radiat. Oncol. Biol. Phys..

[8] Zöller B, Ji J, Sundquist J, Sundquist K (2012). Risk of coronary heart disease in patients with cancer: A nationwide follow-up study from Sweden. Eur. J. Cancer.

[9] Handy CE (2018). Synergistic opportunities in the interplay between cancer screening and cardiovascular disease risk assessment: Together we are stronger. Circulation.

[10] Koelwyn GJ (2020). Myocardial infarction accelerates breast cancer via innate immune reprogramming. Nat. Med..

[11] Koene RJ, Prizment AE, Blaes A, Konety SH (2016). Shared risk factors in cardiovascular disease and cancer. Circulation.

[12] Bertero E, Canepa M, Maack C, Ameri P (2018). Linking heart failure to cancer: Background evidence and research perspectives. Circulation.

[13] Ridker PM (2017). Antiinflammatory therapy with canakinumab for atherosclerotic disease. N. Engl. J. Med..

[14] Prizment AE (2013). Plasma C-reactive protein, genetic risk score, and risk of common cancers in the Atherosclerosis Risk in Communities study. Cancer Causes Control.

[15] Bray F (2018). Global cancer statistics 2018: GLOBOCAN estimates of incidence and mortality worldwide for 36 cancers in 185 countries. CA Cancer J. Clin..

[16] Baade PD, Fritschi L, Eakin EG (2006). Non-cancer mortality among people diagnosed with cancer (Australia). Cancer Causes Control.

[17] Meijers WC (2018). Heart failure stimulates tumor growth by circulating factors. Circulation.

[18] Kenzik KM (2018). New-onset cardiovascular morbidity in older adults with stage I to III colorectal cancer. J. Clin. Oncol..

[19] Dieter RS (2003). Lower extremity peripheral arterial disease in hospitalized patients with coronary artery disease. Vasc. Med..

[20] Welten GM (2008). Long-term prognosis of patients with peripheral arterial disease: A comparison in patients with coronary artery disease. J. Am. Coll. Cardiol..

[21] Colantonio LD (2020). Atherosclerotic risk and statin use among patients with peripheral artery disease. J. Am. Coll. Cardiol..

[22] Caro J, Migliaccio-Walle K, Ishak KJ, Proskorovsky I (2005). The morbidity and mortality following a diagnosis of peripheral arterial disease: Long-term follow-up of a large database. BMC Cardiovasc. Disord..

[23] Suárez C (2010). Influence of polyvascular disease on cardiovascular event rates: Insights from the REACH Registry. Vasc. Med..

[24] Kitsis RN, Riquelme JA, Lavandero S (2018). Heart disease and cancer: Are the two killers colluding?. Circulation.

[25] Malmborg M (2018). Incidence of new onset cancer in patients with a myocardial infarction: A nationwide cohort study. BMC Cardiovasc. Disord..

[26] Blaes AH, Shenoy C (2019). Is it time to include cancer in cardiovascular risk prediction tools?. Lancet.

[27] Liu D (2019). Prevalence and prognosis significance of cardiovascular disease in cancer patients: A population-based study. Aging.

[28] Mehta LS (2018). Cardiovascular disease and breast cancer: Where these entities intersect: A scientific statement from the American heart association. Circulation.

[29] Wilson PW (1998). Prediction of coronary heart disease using risk factor categories. Circulation.

[30] Budoff MJ (2006). Assessment of coronary artery disease by cardiac computed tomography: A scientific statement from the American Heart Association Committee on Cardiovascular Imaging and Intervention, Council on Cardiovascular Radiology and Intervention, and Committee on Cardiac Imaging, Council on clinical cardiology. Circulation.

[31] Perlstein TS, Creager MA (2009). The ankle-brachial index as a biomarker of cardiovascular risk. Circulation.

[32] Simon A, Megnien JL, Chironi G (2010). The value of carotid intima-media thickness for predicting cardiovascular risk. Arterioscler. Thromb. Vasc. Biol..

[33] Hemann BA, Bimson WF, Taylor AJ (2007). The Framingham Risk Score: An appraisal of its benefits and limitations. Am. Heart Hosp. J..

[34] Tini G, Sarocchi M, Ameri P, Arboscello E, Spallarossa P (2019). The need for cardiovascular risk factor prevention in cardio-oncology. JACC Heart Fail..

